# De-epithelialization of porcine tracheal allografts as an approach for tracheal tissue engineering

**DOI:** 10.1038/s41598-019-48450-4

**Published:** 2019-08-19

**Authors:** Fabio G. Aoki, Ratna Varma, Alba E. Marin-Araujo, Hankyu Lee, John P. Soleas, Alexander H. Li, Kayla Soon, David Romero, Henrique T. Moriya, Siba Haykal, Cristina Amon, Thomas K. Waddell, Golnaz Karoubi

**Affiliations:** 1 Latner Thoracic Surgery Research Laboratories, Division of Thoracic Surgery, University Health Network, 101 College St., Toronto, ON M5G 1L7 Canada; 2 Institute of Biomaterials and Biomedical Engineering, University of Toronto, 164 College Street, Toronto, Ontario M5S 3G9 Canada; 3 Department of Mechanical and Industrial Engineering, University of Toronto, 5 King’s College Road, Toronto, Ontario M5S 3G8 Canada; 4 Biomedical Engineering Laboratory, University of Sao Paulo, Escola Politecnica, Av. Prof. Luciano Gualberto 380, Sao Paulo, SP 05508-010 Brazil

**Keywords:** Regenerative medicine, Tissue engineering

## Abstract

Replacement of large tracheal defects remains an unmet clinical need. While recellularization of acellular tracheal grafts appeared to be a viable pathway, evidence from the clinic suggests otherwise. In hindsight, complete removal of chondrocytes and repopulation of the tracheal chondroid matrix to achieve functional tracheal cartilage may have been unrealistic. In contrast, the concept of a hybrid graft whereby the epithelium is removed and the immune-privileged cartilage is preserved is a radically different path with initial reports indicating potential clinical success. Here, we present a novel approach using a double-chamber bioreactor to de-epithelialize tracheal grafts and subsequently repopulate the grafts with exogenous cells. A 3 h treatment with sodium dodecyl sulfate perfused through the inner chamber efficiently removes the majority of the tracheal epithelium while the outer chamber, perfused with growth media, keeps most (68.6 ± 7.3%) of the chondrocyte population viable. De-epithelialized grafts support human bronchial epithelial cell (BEAS-2B) attachment, viability and growth over 7 days. While not without limitations, our approach suggests value in the ultimate use of a chimeric allograft with intact donor cartilage re-epithelialized with recipient-derived epithelium. By adopting a brief and partial decellularization approach, specifically removing the epithelium, we avoid the need for cartilage regeneration.

## Introduction

Tracheal replacement is necessary when the extent of the damaged organ is more than 50% of the length in adults or 30% in children^1^. For instance, long tracheal stenosis and neoplastic cases can extend widely through the trachea. Several sources for tracheal repair and replacement have been evaluated over the last few decades. These can be divided into: the use of autologous^2^ and nonviable tissues^3^, foreign materials^4^, and naturally-derived biological acellular scaffolds^5^.

Complete decellularization of donor tracheal grafts has received the most attention^6–8^, theoretically attractive as it removes immunogenic cellular material while maintaining the structure and composition of the extracellular matrix (ECM). While there has been extensive research on decellularization protocols^5,9–12^, *in vivo* decellularized scaffolds have faced challenges with airway stenosis due to compromised cartilage^8,13^. Attempts at recellularization have not been successful^9^ and recent clinical studies have brought into doubt the feasibility of this approach. Perhaps the possibility of adequate repopulation of a completely decellularized graft was unrealistic. Specifically, penetration of the chondroid matrix and reconstitution of the required chondrocytes remains a formidable challenge^14–16^.

Given the low immunogenicity of the tracheal cartilage^17–21^, complete decellularization of tracheal grafts may not be required. This is a completely different approach in which a hybrid graft is generated after removal of only the epithelium, with proof-of-concept studies suggesting clinical applicability^15,20,22^. Current partial decellularization approaches, however^19,21^, are lengthy, requiring up to 48 h of detergent treatment, do not necessarily sustain cartilage viability^21^, and are not characterized with respect to mechanical properties.

**Figure 1:**
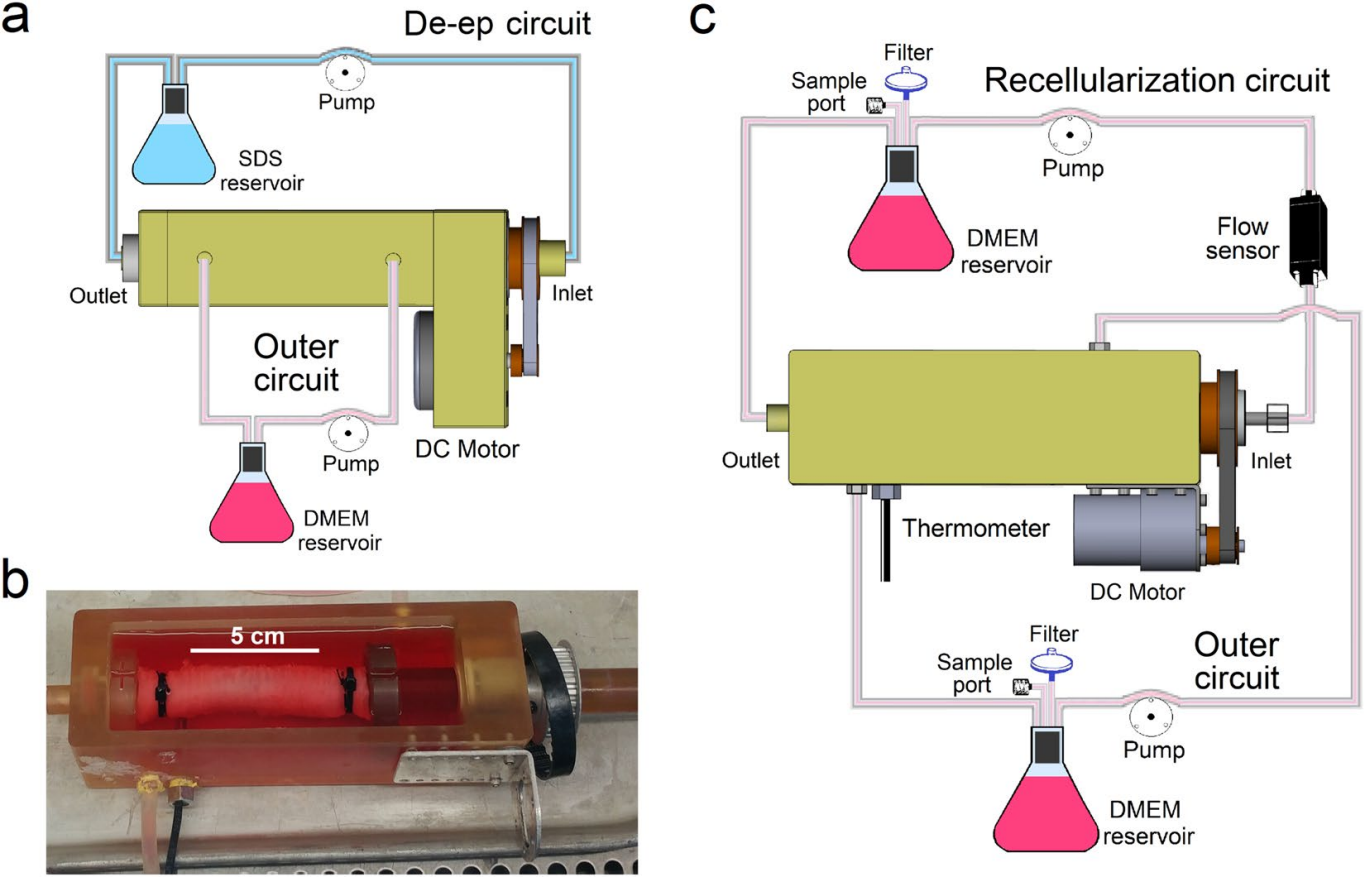
Double-chamber bioreactor configuration during De-epithelialization (De-ep) and Recellularization. (**a**) Schematic representation of the De-ep process in De-ep bioreactor system. (**b**) Image of a de-epithelialized long segment porcine tracheal graft attached in the recellularization bioreactor system. (**c**) Schematic representation of the recellularization process in recellularization bioreactor system showing the addition of sample ports and filters, flow sensor and thermometer to the bioreactor system used for De-ep.

Here, we present a novel, clinically-relevant, partial decellularization approach to generate hybrid tracheal grafts (Fig. 1). Specifically, we use a short (3 h) detergent-based treatment with sodium dodecyl sulfate (SDS) to remove only the epithelium and maintain the structural integrity of the donor grafts while keeping the majority of cartilage alive. The entire process is completed in a bioreactor setting, which is then used for re-epithelialization.

## Results

### Tracheal de-epithelialization & morphological evaluation

Our double chamber perfusion bioreactor was set-up and assembled as previously described^23^. De-epithelialization steps are summarized in Supplementary Table S1. During the de-epithelialization phase (Fig. 1a), inner and outer chambers connecting reservoirs and pumps allowed the fluid to flow throughout the duration of the protocol. The inner circuit was used to perfuse 1% SDS solution for 3 h to remove the cells from the lumen while the outer circuit was used to support the chondrocytes. The time of treatment with SDS for partial decellularization was optimized according to histological and morphometric analysis (Supplementary Figs S1 and S2). Selection of the 3 h de-epithelialization time was based on balancing complete removal of epithelium and cellular debris with adverse effects of detergent on tissue and shows that a 3 h treatment with 1% SDS efficiently removes the majority of the epithelium. At 1 h, however, remnants of the epithelium are still observed. Further increasing time of SDS treatment (5, 12, 24 and 48 h) did not increase the efficacy of epithelial cell removal from the tracheal lumen.

**Figure 2:**
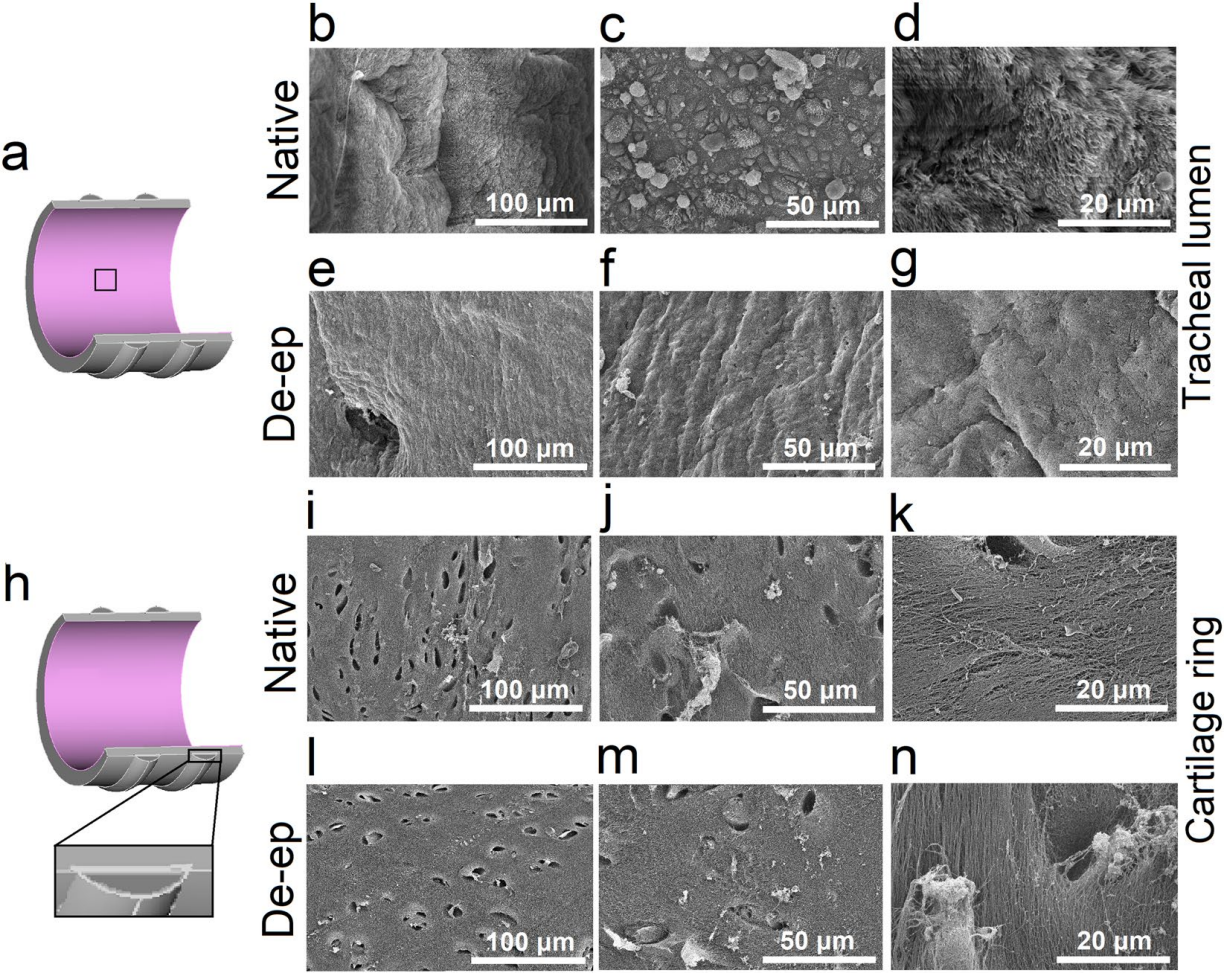
Scanning electron microscopy (SEM) images of tracheal lumen and cartilage cross-section of Native and De-epithelialized (De-ep) samples. (**a**) Representation of the trachea with the tracheal lumen *en face* (box insert). (**b**–**d**) SEM images of the luminal surface of Native tracheal samples (n = 3). (**e**–**g**) Luminal surface of De-ep tracheal samples (n = 3). (**h**) Schematic representation of the cartilage ring cross-section (box insert). (**i**–**k**) SEM images of the cross-section of Native tracheal samples (n = 3). (**l**–**n**) Cross-section of De-ep tracheal samples (n = 3).

Scanning electron microscopy (SEM) images of native tracheal luminal surface (Fig. 2a) depicted an intact epithelium (Fig. 2b–d). In contrast, SEM images of de-epithelialized tracheae showed near-complete removal of the epithelium, leaving behind a smooth, intact basement membrane (Fig. 2e–g). Importantly, SEM images of the tracheal cartilage in cross-section (Fig. 2h) did not show major differences between the native (Fig. 2i–k) and de-epithelialized (Fig. 2l–n) samples, suggesting that the de-epithelialization process does not negatively impact the cartilage morphology.

### Characterization of extracellular matrix components in de-epithelialized grafts

**Figure 3:**
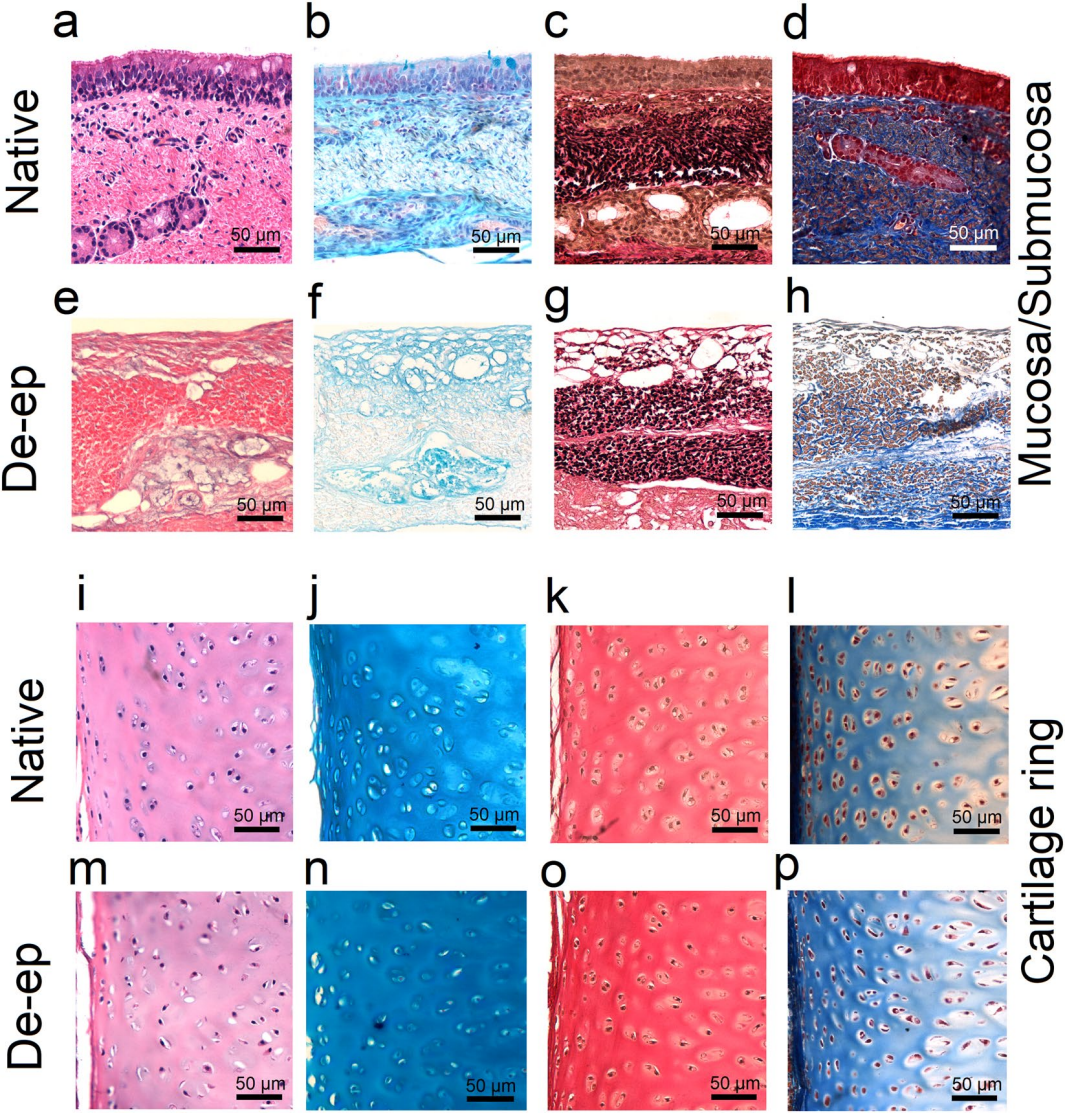
Histological evaluation of Native and De-epithelialized (De-ep) tracheal samples. Representative microscopy images of Native and De-ep tracheal samples (n = 3) showing (**a**,**e**,**i**,**m**) haematoxylin & eosin (H&E) staining, (**b**,**f**,**j**,**n**) Alcian blue staining for glycosaminoglycans, (**c**,**g**,**k**,**o**) elastin staining for elastic fibers, and (**d**,**h**,**l**,**p**) Masson’s trichrome staining for collagen fibers. Mucosa/Submucosa samples of (**a**–**d**) Native and (**e**–**h**) De-ep tracheal grafts. Cartilage samples of (**i**–**l**) Native and (**m**–**p**) De-ep tracheal grafts.

**Figure 4:**
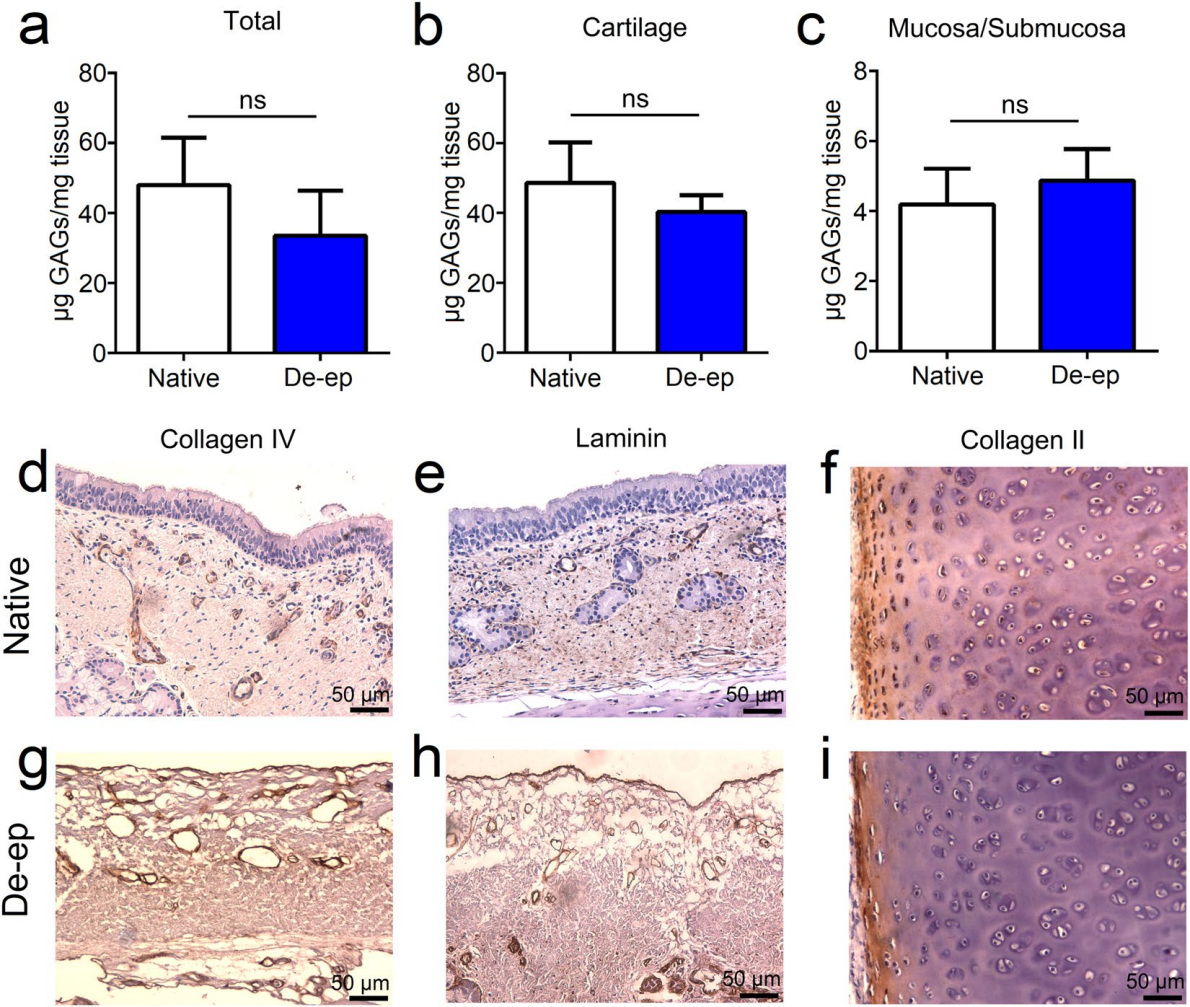
Quantification of sulfated glycoaminoglycans (sGAGs) and immunohistochemical evaluation of Native and De-epithelialized (De-ep) samples. (**a**–**c**) Quantification of sGAGs in Native and De-ep samples. Quantified sGAGs (**a**) in Total (intact) Native (n = 7) and De-ep (n = 5) tracheal samples containing both Cartilage and Mucosa/Submucosa; (**b**) in isolated Cartilage from Native (n = 5) and De-ep (n = 5) tracheal samples; and (**c**) in isolated Mucosa/Submucosa from Native (n = 6) and De-ep (n = 6) tracheal samples. Bars represent mean ± standard deviation (ns, p > 0.05). (**d**–**f**) Native and (**g**–**i**) De-ep samples. (**d**–**i**) are representative (n = 3) immunohistochemistry images for collagen type IV, laminin and collagen type II, respectively.

To confirm that de-epithelialization efficiently removes the epithelium while maintaining extracellular membrane (ECM) components, de-epithelialized samples were analyzed qualitatively for the presence of cells, glycosaminoglycans (GAGs), elastin content and collagen (Fig. 3). Histological evaluation of native tracheal samples showed a pseudostratified columnar epithelium facing the lumen with submucosal glands deep in the tissue (Fig. 3a). In de-epithelialized samples, the pseudostratified epithelium was not present (Fig. 3e). Although in most cases de-epithelialization resulted in the removal of the cell nuclei from the submucosa, in some samples, cellular remnants were still present in the submucosa (Supplementary Fig. S3). In contrast to the differences observed between native and de-epithelialized samples with respect to the presence of epithelium, the cartilaginous rings of the de-epithelialized samples had approximately the same structural patterns when compared with native samples, with no differences in the profile of the fibers observed (Fig. 3i,m). Alcian blue staining for GAGs showed no observable differences between native and de-epithelialized samples (Fig. 3b,f,j,n). In accordance with the qualitative results, the quantification of sulfated glycosaminoglycans (sGAGs) demonstrated no significant differences in sGAGs content of native and de-epithelialized samples (Fig. 4a–c). De-epithelialization in the bioreactor also preserved elastin (Fig. 3c,g,k,o) and collagen (Fig. 3d,h,l,p) with no major differences between de-epithelialized and native samples. Further evaluation of basement membrane proteins using immunohistochemical analysis confirmed the presence of both collagen type IV (Fig. 4d,g) and laminin (Fig. 4e,h) in the basement membrane of the de-epithelialized samples. Moreover, collagen type II staining (a hyaline cartilage collagen) was also similar between the native and de-epithelialized tracheal samples (Fig. 4f,i).

### Mechanical analysis

To determine if our process maintains structural integrity, we evaluated the effect of the bioreactor-based de-epithelialization process on mechanical properties of tracheal grafts. Specifically, we assessed and compared tracheal hysteresis, compliance and energy loss in native versus de-epithelialized grafts. Hysteresis is linked to the viscoelastic behavior of a biological material^24^ and can be interpreted as the dissipated energy during a load-unload cycle^25^. A higher hysteresis means that the energy used to expand the trachea is not fully recovered during deflation. As the name implies, viscoelastic materials present viscous (or dissipative) and elastic (or energy storage) behaviors, and the higher the hysteresis, the greater the dissipative component of the material. We measured both the area under the pressure-volume (PV) curve as well as the energy loss as a measure of tracheal viscoelasticity.

**Figure 5:**
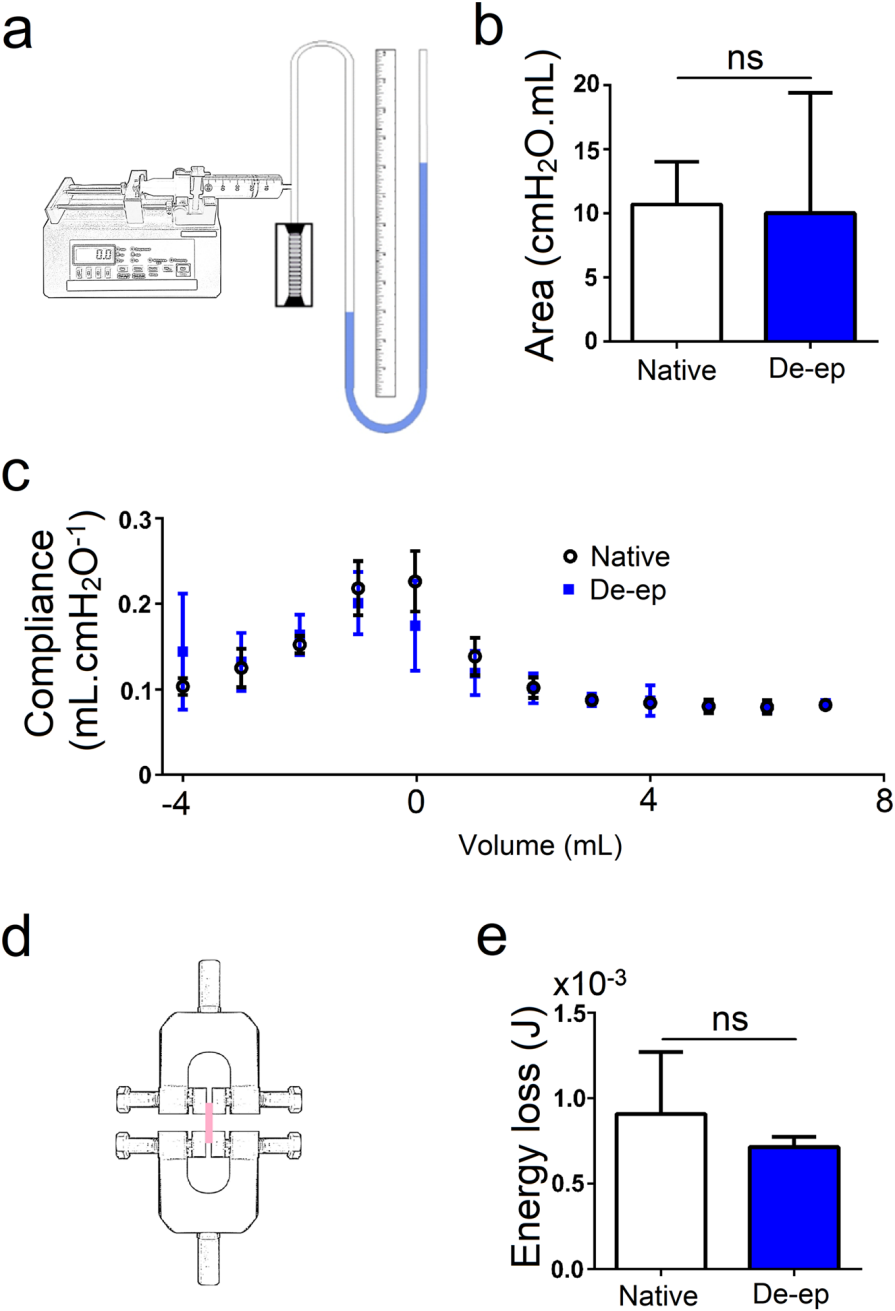
Mechanical analysis of Native and De-epithelialized (De-ep) samples. (**a**) Schematics of the pressure-volume (PV) curve experiment. (**b**) Area under the PV curve and (**c**) compliance measured every two volume-steps of fresh Native tracheal grafts (n = 5) and De-ep grafts (n = 7). (**d**) Schematics of the clamped cartilaginous ring. (**e**) Calculated energy loss from the fifth cycle of Native cartilage rings and De-ep samples (n = 3). Bars represent mean ± standard deviation (ns, p > 0.05).

The configuration of the system to obtain the PV curve data and compliance is depicted in Fig. 5a. The area under the PV curve of native grafts (5.1 cm ± 0.4 cm, mean ± standard deviation) and de-epithelialized grafts (5.1 cm ± 0.5 cm) is presented in Fig. 5b. The de-epithelialization process did not significantly alter (p = 0.880) the area under the curve as compared to native samples. This suggests that the viscoelastic property of the tissue graft is not significantly changed by the de-epithelialization process. The compliance, measured every two-volume steps of 1 mL for native and de-epithelialized grafts during deflation (Fig. 5c), also demonstrated no statistical differences (p > 0.05). Similarly, there were no significant differences in energy loss calculated for isolated cartilage rings of native and de-epithelialized samples (p = 0.700; Fig. 5d,e).

### Chondrocyte viability

**Figure 6:**
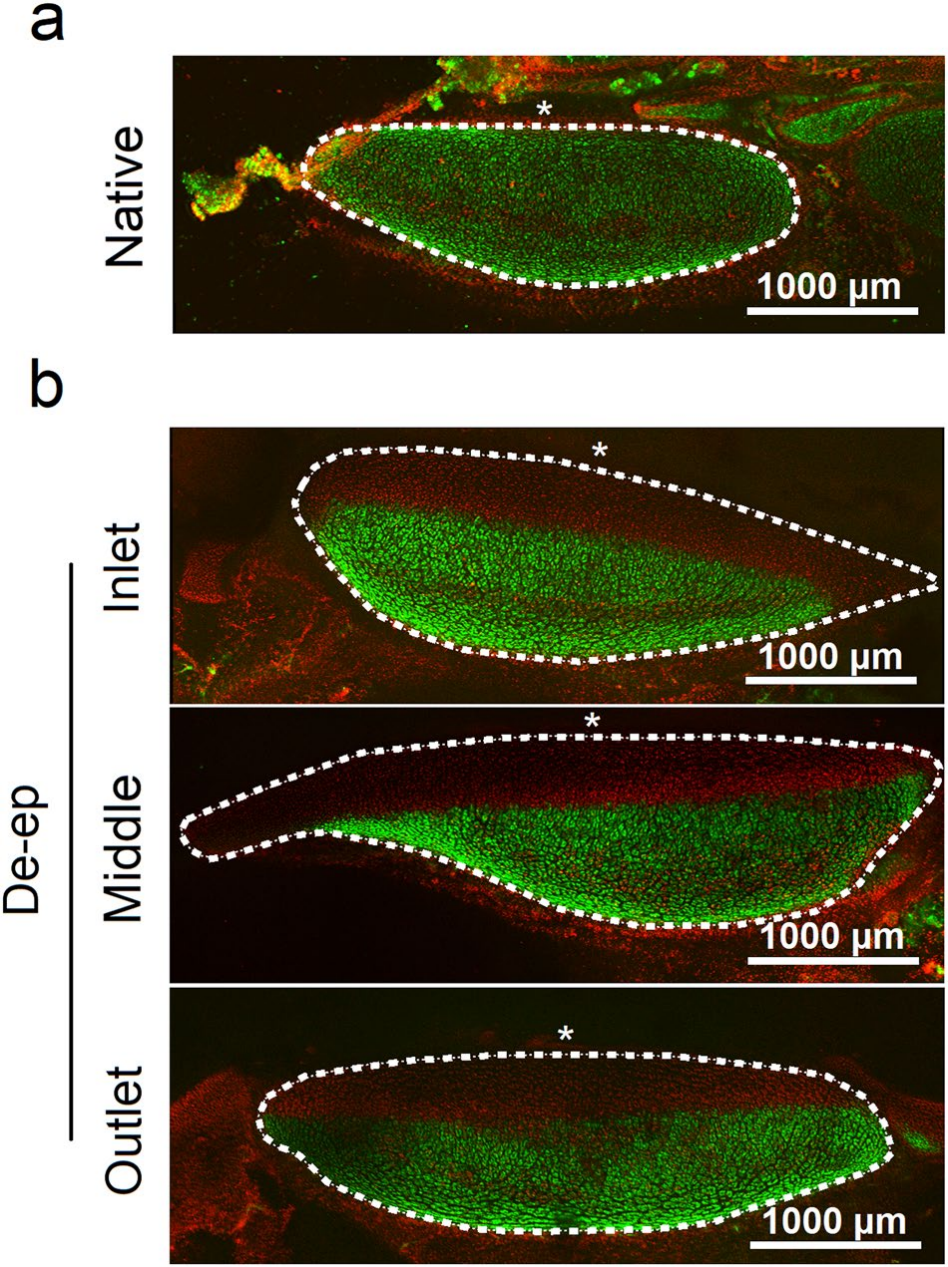
Chondrocyte viability in Native and De-epithelialized (De-ep) samples. Confocal microscopy images depicting calcein-AM for live (green) and ethidium homodimer-1 for dead (red) cells in cross-sections of cartilage rings (marked as the area within the white dotted line) in (**a**) Native samples (n = 3; 3 sections per sample), and (**b**) representative regions (inlet, middle and outlet; 4 sections per region) of De-ep samples (n = 4). White asterisks mark the position of the tracheal lumen.

To validate our microscopic and histological evaluation of tracheal cartilage integrity, we assessed chondrocyte viability immediately following our de-epithelialization protocol. Cross-sectional slices of cartilage rings were used to assess cell viability. Viability was confirmed via intracellular esterase activity using calcein-AM green fluorescent staining. Live chondrocytes were evident throughout the native cartilage rings Fig. 6a. De-epithelialized grafts were evaluated and chondrocyte viability was quantified across three different regions (inlet, middle, and outlet; 4 sections per region) of the samples (Fig. 6b).

Quantification of the viable cartilage area (green) compared to the total area (green and red) showed that the de-epithelialization process maintains cartilage viability at 68.6 ± 7.3%; with no statistically significant differences in chondrocyte viability between the three regions (inlet (76.8 ± 8.86%); middle (62.7 ± 17.3%); outlet (66.5 ± 13.8%); Bartlett’s test for equality of variance: p = 0.578; ANOVA test for significance: p = 0.366). While the protocol preserved the majority of the chondrocytes, we observed a layer of necrotic chondrocytes facing the tracheal lumen (Fig. 6b), which we believe could be due to closer proximity to the SDS detergent solution used during the procedure or residual SDS remaining in the graft. Cell death could also be attributed to residual SDS remaining in the graft. Our protocol includes a 30 min wash with Triton X-100 solution, known to efficiently remove soluble SDS^26,27^. However, it is likely that some SDS has been immobilized within the graft and escapes removal^27,28^. Taken together, these results showed that the bioreactor-based de-epithelialization process preserves the majority of the tracheal cartilage.

### Cell seeding and repopulation of de-epithelialized grafts

**Figure 7:**
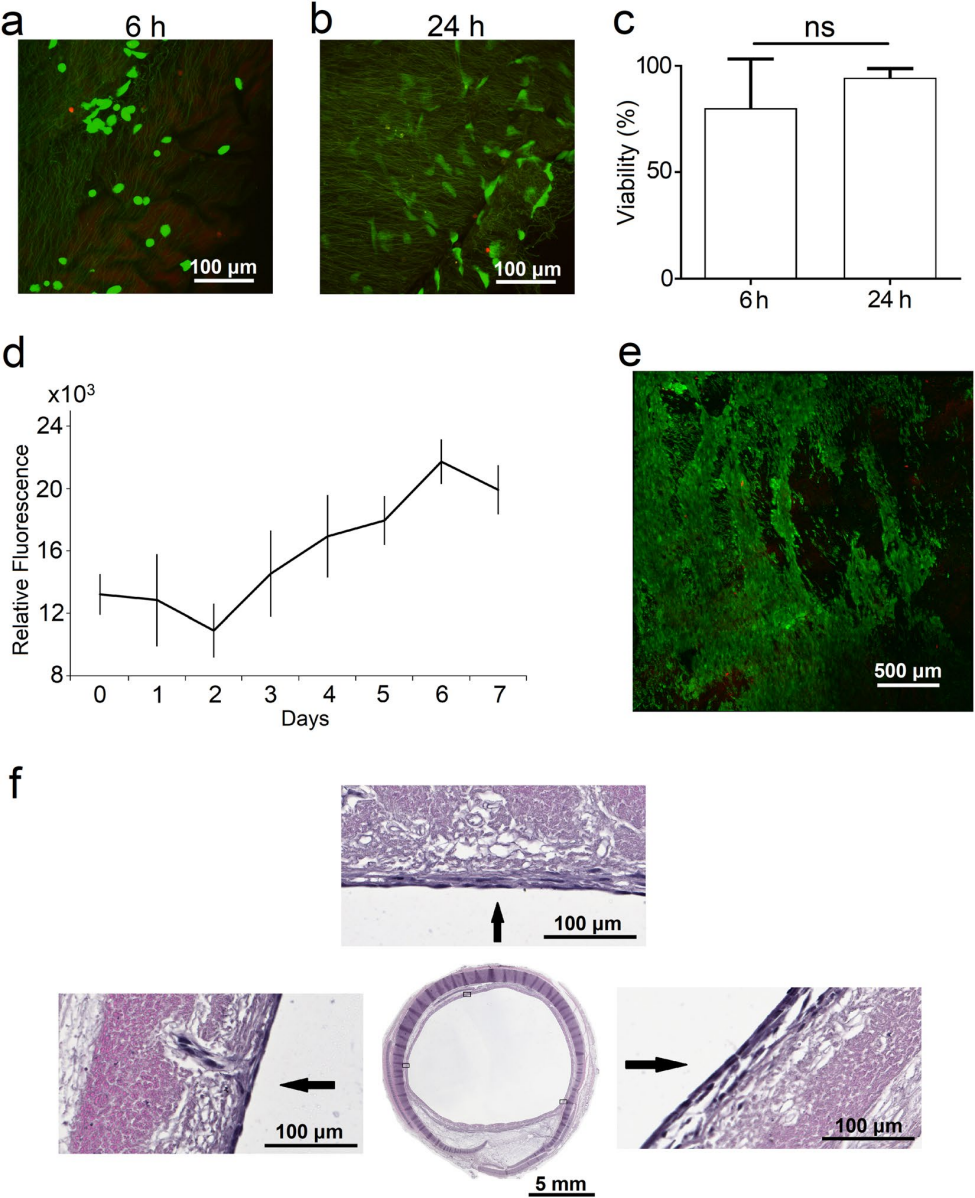
Recellularization of De-epithelialized (De-ep) tracheal grafts. Confocal microscopy images of peeled Mucosa/Submucosa from De-ep tracheal samples seeded with human bronchial epithelial cells (BEAS-2Bs) at (**a**) 6 h and (**b**) 24 h after cell seeding (n = 4). Live cells are depicted in green (calcein-AM) and dead cells are shown in red (ethidium homodimer-1). (**c**) Cell viability (n = 4; 3–7 quantified fields per sample) of BEAS-2Bs reported as percentage (%) of live cells per total cells (bars are mean ± standard deviation; ns, p > 0.05). (**d**) Metabolic cell activity of BEAS-2Bs in bioreactor during a 7 day culture period (n = 3; data represented as mean ± standard deviation). (**e**) Representative (n = 3; 3 fields per sample) confocal microscopy image of recellularized tracheal lumen following a 7 day culture period in the bioreactor. Live cells are depicted in green (calcein-AM) and dead cells are shown in red (ethidium homodimer-1). (**f**) Brightfield microscopy images showing haematoxylin and eosin (H&E) staining of a representative tracheal graft recellularized with BEAS-2Bs following a 7 day culture period in bioreactor (n = 3).

Recellularization of de-epithelialized grafts was first evaluated in 2D culture via direct cell seeding on peeled submucosa from de-epithelialized tracheal samples. Peeled segments of submucosa were seeded with BEAS-2B cells (human bronchial epithelial cell line; ATCC® CRL-9609™, USA) and analyzed for attachment and viability 6 and 24 h after cell seeding (Fig. 7a,b). Cell viability was quantified as the mean percentage of live cells per total number of cells in a field (3–7 fields per sample). BEAS-2B cell viability was 80.1 ± 22.9% after 6 h and 94.3 ± 4.3% after 24 h. De-epithelialized grafts were able to support BEAS-2B cell attachment and viability at least up to 24 h (Fig. 7c).

For proof-of-concept, de-epithelialized grafts were then recellularized using BEAS-2B cells in our bioreactor system modified for recellularization (Fig. 1b,c). During the recellularization phase, the inner lumen was connected to a perfusion circuit with cell growth media (Fig. 1c). Growth media was circulated continuously for 7 days. Cell metabolism was evaluated daily for 7 days with a resazurin-based assay (Fig. 7d) showing increasing rate of cell metabolic activity after an initial lag period, likely due to a period of cell adaptation in the bioreactor. Calcein-AM green staining after a 7 day period in the bioreactor showed presence of viable epithelium on recellularized grafts (Fig. 7e) and histological evaluation (Fig. 7f) demonstrated an intact cell layer lining the luminal surface.

## Discussion

We present here a clinically relevant system using a short 3 h detergent-based treatment method in a bioreactor setting to remove native epithelium from porcine tracheal grafts. Our approach allows for maintenance of mechanical properties, native architecture, ECM composition and cartilage viability. Furthermore, de-epithelialized grafts re-populated in the bioreactor support airway cell attachment and viability.

While a handful of partial decellularization approaches have been reported^17,19,21^, ours is unique as it is performed in a completely closed system using a double perfusion bioreactor^23^. This has significant clinical translation potential as our approach ultimately enables recellularization to begin immediately following de-epithelialization without any additional manipulation of the graft maintaining sterility and preventing contamination.

Sodium dodecyl sulfate (SDS) has been extensively used for decellularization in various organs and tissues, such as kidney^29^, aorta^30^, heart^26^, heart valves^31^ and lungs^32^. In the trachea, SDS has reportedly been used in two recent protocols both targeting full decellularization of the tracheal graft aiming to remove all cellular material including chondrocytes. Weymann *et al*.^33^ use continuous exposure of pig tracheal grafts to 4% SDS solution over a 3 day protocol. Hung and colleagues^34^ use a more complex multi-cycle protocol to decellularize rabbit trachea. Specifically, they used 3 cycles of a 24 h freeze-dry step followed by a 1 h sonication step in the presence of 1% SDS solution. We found that a 3 h treatment period using a 1% final concentration of SDS was sufficient to remove tracheal epithelium and did not have subsequent detrimental effects on graft structure and composition. Importantly, at this concentration we were able to keep the majority of the cartilage alive. Although the removal of chondrocytes has proven to be difficult irrespective of protocol used^5,8,9^, to date no one has shown convincing evidence that these chondrocytes remain viable after the decellularization process. We did observe a layer of necrotic chondrocytes facing the tracheal lumen, which we believe to be due to closer proximity to the SDS detergent solution used during the procedure.

Cartilage regeneration remains a challenge in the recellularization of decellularized grafts. Our group^23^, amongst others^35^, have used mesenchymal stromal cells (MSCs) in lieu of chondrocytes. MSCs have some advantages such as relative ease of isolation and culture and proliferative capacity^36^, although they may not fully recapitulate the structural and functional properties of the native tracheal cartilage. Moreover, adequate penetration of MSCs (and/or other cell types used for cartilage regeneration) into the cartilage lacunae is very challenging^37^. By keeping the cartilage alive, our approach circumvents the need for cartilage regeneration; therefore, significantly simplifying the recellularization process.

Evaluation of tracheal biomechanics is important in the analysis of decellularized tracheal grafts^38^ and often used as a measure to predict tracheal collapse. Grafts that mimic the native trachea are deemed ideal. Few studies have evaluated the macromechanical properties of fully decellularized tracheal grafts. To this end, we have shown that long treatments with different detergents could both increase or decrease the compliance of tracheal grafts^9^. For evaluation of our de-epithelialized porcine tracheal grafts, we here present an assessment of macromechanics which we define to encompass compliance, hysteresis, and energy loss. As expected, we show that mechanics of long segments and isolated cartilage rings of de-epithelialized tracheal grafts do not present statistically significant differences when compared to native samples.

Qualitative analyses revealed that basement membrane and ECM components such as collagen, elastin, laminin and proteoglycans are preserved after the short protocol of de-epithelialization and the same was observed for the quantitative analyses of sGAGs. For structural integrity and graft function, decellularization approaches must not negatively impact the basement membrane and ECM proteins. In fact, we have previously demonstrated^9^ that decellularization cycles reduce the amount of GAGs. This could contribute to loss of mechanical integrity of the tracheal scaffold^39^.

While other attempts to improve decellularization protocols have shown the gross preservation of ECM after the procedure, the basement membrane seems to be compromised by both the removal of laminin and collagen^11^, and by the apparent rupture of the fibers^40^. Our bioreactor-based approach not only preserves ECM proteins and sGAGs, but the 3 h SDS treatment also preserves the basement membrane. For proof-of-concept, we showed that our de-epithelialized grafts are supportive of BEAS-2B cell attachment, viability, and growth in the bioreactor for up to 7 days.

Our study is not without limitations. Even though our approach removes the epithelium, we do observe glands that are not fully decellularized closer to the cartilage and also in the membranous regions. This was anticipated as we have previously shown that alloimmunogenic components (major histocompatibility complex I and II) were still present in submucosal glands even after 17 cycles of decellularization (one protocol lasting ~35 days and the other more than 3 months)^41^. While beyond the scope of this study, future studies evaluating the fate of the de-epithelialized grafts *in vivo* will determine whether further approaches to remove remaining glands will be necessary.

Another limitation of our study is the need for long-term re-epithelialization in the bioreactor with a more relevant cell type. Our current repopulation studies were conducted with BEAS-2B cells which are not necessarily representative of primary cells. Future studies will include re-epithelialization of de-epithelialized tracheal grafts with human tracheal epithelial cells (HTECs). As HTECs take approximately ~14–21 days for maturation, re-epithelialization experiments within the bioreactor will need to be conducted for several weeks, which is testable using our current design.

This study presents a novel clinically relevant protocol using a bioreactor-based method of de-epithelialization for the development of tracheal grafts. The short protocol has shown to preserve both structural and mechanical integrity of the graft while keeping viable cartilage. The preservation of the basement membrane facilitates cell attachment, viability and proliferation of cells during repopulation. Our approach reduces the time of decellularization while maintaining the viability of the cartilage, and subsequently enables cell attachment and repopulation. Thus, it can ultimately have wide implications in tissue engineering approaches for tracheal replacement.

## Methods

### Animals

All studies were approved by the Animal Care Committee of the Toronto General Research Institute. Humane care was provided to all animals in conformity with the “Principles of Laboratory Animal Care” defined by the National Society for Medical Research and the “Guide for the Care of Laboratory Animals” issued by the National Institutes of Health. Tracheal samples were collected from 3-months old outbred male Yorkshire pigs (n = 26, 28–35 kg) used in transplant studies. The detailed protocol of the surgery has been described previously^42^. Briefly, pigs were anesthetized using an intramuscular (i.m.) injection of a mixture of ketamine (25 mg/kg), atropine (0.04 mg/kg), and midazolam (0.15 mg/kg). Prior to harvest of the grafts, pigs were maintained at 5% isoflurane. The animals were sacrificed using an overdose of isoflurane and propofol. Tracheae were collected after confirmation of death.

### Tracheal graft harvest

Prior to de-epithelialization, tracheal grafts were harvested, collected in the operating room under sterile conditions, and stored in Hank’s balanced salt solution (HBSS) solution on ice. The storage solution consists of HBSS containing 2% Bovine Serum Albumin (BSA) and an antibiotic-antimycotic solution which included the following: fluconazole (4 µg/mL), colistimethate (5 µg/mL), imipenem/cilastatin (25 µg/mL), ceftazidime (154 µg/mL), penicillin (200 U/mL), streptomycin (200 µg/mL), amphotericin B (0.5 µg/mL) and gentamicin (50 µg/mL).

### Bioreactor de-epithelialization and re-epithelialization protocol

Our double chamber bioreactor was set-up and assembled as previously described^23^. The de-epithelialization protocol is summarized in Supplementary Table S1. During de-epithelialization (37 °C), two independent sets of silicone tubing in each chamber (inner and outer) were connected to reservoirs (140 mL) and peristaltic pumps (Variable flow, VWR Pumps, Canada) and allowed fluid to circulate in the tubing throughout the duration of the protocol (Fig. 1a). The inner chamber was filled with 1% SDS (w/v) in deionized water (diH_2_O) through a reservoir (75 mL) for 3 h to remove the cells from the lumen while the tracheal scaffolds were being rotated (8 rpm). At the same time, the outer chamber was filled with growth media, Dulbecco’s modified Eagle’s medium (DMEM, Gibco, USA), supplemented with 10% (v/v) fetal bovine serum (FBS; Gibco, USA) and 1% (v/v) penicillin-streptomycin (Gibco, USA)) to support the chondrocytes. The lumen was then washed for 40 min with diH_2_O (240 mL), 30 min with 1% Triton X-100 in diH_2_O (140 mL), and 30 min with 140 mL of phosphate buffered saline (PBS). At the end of the protocol, the motor was disengaged, the bioreactor was disassembled in a biosafety cabinet, and tissue samples were harvested for further analysis.

For recellularization, a modified bioreactor system was used which allowed for longer term recellularization. Modifications included addition of sample ports for media changes and sample collection for metabolic activity assays; a thermometer inside the bioreactor to precisely control temperature over the 7 day culture period; and a flow sensor. De-epithelialized tracheal grafts were decontaminated in HBSS supplemented with 2% (w/v) bovine serum albumin (BSA) and a broad spectrum cocktail solution for 48 h (with solution change and luminal scraping every 24 h, at 4 °C) on a rocking platform (30 rpm). The de-epithelialized tracheal graft was then reattached to an autoclaved recellularization bioreactor and a suspension (1 mL) of BEAS-2Bs (~1 × 10^6^ cells/cm^2^ of tracheal lumen) was injected into the tracheal lumen. In the recellularization phase (Fig. 1c), cells were allowed to adhere for 2 h under static conditions with a rotation of 5 rpm, and subsequently cultured in growth media under unidirectional flow (1.5 mL/min) for the rest of the experiment. During the recellularization process the media circulating through the lumen (inner circuit; 30 mL in total volume) was changed every 24 h and half of the media bathing the outside of the trachea (outer circuit; 250 mL in total volume) was changed every 48 h.

### Cell metabolic activity assay

Metabolic cell activity was evaluated by a resazurin-based assay as per manufacturer instructions (Presto Blue, Invitrogen). Briefly, a solution (20 mL) containing 1:20 (v/v) Presto Blue/DMEM with 10% FBS was prepared and a 1.5 mL volume was separated for use as control. The remaining reagent was perfused (1.5 mL/min at 37 °C) in the bioreactor through the lumen of the seeded scaffold for 1 h and a sample was obtained for fluorescence analysis at 560 nm (Cytation™ 5, BioTek Instruments) on a 24-well plate.

### Histology and immunohistochemistry

Tracheal samples were fixed in 10% formalin neutral buffered solution overnight, and transferred to 70% ethanol until paraffin embedding. Paraffin blocks were then sectioned (5 µm), and deparaffinized in xylene. Sample slides were stained for histology (haematoxylin and eosin (H&E), Alcian blue, elastin, and Masson’s trichrome) and immunohistochemistry (antibodies against collagen types II and IV, and laminin).

H&E staining was performed according to a routine protocol used in the laboratory^9^. Alcian blue (NovaUltra™, IHC World, USA), Elastic (Sigma-Aldrich, USA) and Masson’s trichrome (Sigma-Aldrich, USA) staining were performed using respective kits according to manufacturers’ protocols. Immunohistochemistry samples were evaluated for collagen type II (dilution 1:1200; ab34712; Abcam, USA), collagen type IV (dilution 1:1500; ab6586; Abcam, USA), and laminin (dilution 1:50; ab11575; Abcam, USA) using the RTU Vectastain® kit (Vector Laboratories, Canada). Antigen retrieval was performed via heat induction in 1x citrate buffer (Sigma, USA), followed by an incubation in 3% H_2_O_2_ (v/v) at room temperature (5 min) and a PBS wash. Normal horse serum 2.5% (v/v) was used for 30 min and the primary antibody was incubated overnight at 4 °C using the aforementioned dilutions. A 30-minute biotinylated secondary antibody incubation was followed by a 5-minute PBS wash, a 30-minute incubation in RTU Vectastain® ABC (avidin-biotin complex, Vector Laboratories, Canada) Reagent, and another 5-minute PBS wash. Then, 30 µL of ImmPACTTM DAB Chromogen was diluted in 1 mL of ImmPACTTM DAB Dilutent and placed onto each slide. Samples were counterstained in hematoxylin solution Gill’s n° 2, washed in tap water, dehydrated in xylene, cleared, and mounted.

### Scanning electron microscopy (SEM)

Native and de-epithelialized samples were fixed overnight in a solution containing 2% formaldehyde and 0.5% glutaraldehyde in PBS at 4 °C. Samples were washed for 3 × 15 min in PBS to remove unfixed aldehydes and post-fixed in 1% osmium tetroxide solution for 30 min, followed by a secondary wash of 2 × 20 min in PBS. Serial ethanol dilutions (2 × 15 min in 30%, 2 × 20 min in 50%, 2 × 30 min in 70%, 2 × 45 min in 90%, 2 × 1 h in 100%, and overnight in 100% at 4 °C) were performed to dehydrate the samples. Samples were then critical point dried (purge phase of 30 min; Tousimis Autosamdri 810, USA), mounted with carbon colloid paint on scanning electron microscope stubs and gold-palladium sputter coated (2 min; Polaron SC7640 Sputter Coater; Quorum Technologies, Canada). Then, the stubs containing the samples were placed on a scanning electron microscope (S-3400, Hitachi High-Technologies, Canada) at 10 kV.

### Biochemical assay

The quantification of sulfated glycosaminoglycans (sGAGs) was performed using an assay kit (BlyscanTM, Biocolor, UK). Snap-frozen samples were thawed at room temperature and sample rings were split into three groups: intact (cartilage and mucosa/submucosa), cartilage and mucosa/submucosa. Samples weighing 50 mg were digested in 1.5 mL Eppendorf tubes containing 1 mL of papain extraction reagent (5 µM papain, 0.1 M sodium acetate, 10 mM EDTA, 5 mM L-cysteine HCl, 0.2 M sodium phosphate monobasic, 0.2 M sodium phosphate dibasic, pH 6.4) and kept overnight in a water bath at 65 °C with occasional vortexing. Samples were then centrifuged at 4500 rpm and 750 µL of supernatant of each tube was placed into a new Eppendorf tube. An additional 750 µL of papain extraction reagent was added to the remaining 250 µL of digested sample to complete the digestion. Digested samples were vortexed and all paired samples were again mixed (total of 1,750 µL of digested solution) into 15 mL tubes, vortexed, centrifuged at 4500 rpm at 4 °C for 20 min. The supernatants from the digested samples were placed into 1.5 mL Eppendorf tubes and diH_2_O was added to complete a volume of 100 µL. 1 mL of dye reagent (Blyscan Dye Reagent, BlyscanTM assay kit, Biocolor, UK) was added and the tubes were vortexed and stirred in an orbital shaker at 300 rpm at 37 °C for 30 min to allow the sGAG-Dye binding. Samples were then centrifuged at 4500 rpm for 20 min and the supernatant was subsequently discarded. Then, 1 mL of dissociation reagent (Blyscan Dissociation Reagent, BlyscanTM assay kit, Biocolor, UK) was added to the tubes and vortexed until no solids could be seen. Triplicates of 200 µL were transferred into 96-well plate and the absorbance readings were measured at 656 nm on the plate reader (CytationTM 5, BioTek, USA). Standard curves (r^2^ > 0.99; Supplementary Fig. S4) were performed for each experiment.

### Pressure-volume relationships

The tracheae were attached to our previously described system^9^. The tubing attached to a three-way stop-cock was connected to a water column to determine the pressure of the system. Once attached, the tracheal graft was pre-cycled ten times in order to approach a more uniform hysteresis^8^. The volume was obtained by the position of the plunger in the syringe. Next, a quasi-static pressure-volume curve was performed with 1 mL volume steps until stabilization of the pressure, with injected volume ranging from 1 to 6–8 mL. To avoid dehydration, tracheal samples were constantly hydrated with the same solution that they had been stored in. The area of the PV curve was calculated in a mathematical programming environment (Matlab, The MathWorks, USA) using the trapezoidal rule.

### Tensile testing

Tensile testing protocol was performed as described in our previous study^43^. To evaluate the area under the load versus displacement curve of the cartilage rings, a tensile test instrument (Model 840LE2, TestResources Inc., USA) was used to perform uniaxial tests. The initial length of cartilage rings was approximately 30 × 5 mm (length × width) and sand-paper was used to increase the grip. Tracheal samples were stretched periodically for 5 cycles at a stretch speed of 3 mm/min and 10% strain (similar to that performed by^44^) in a triangular wave form. Cartilage samples were kept in PBS at room temperature until time of experiment. The samples were sprayed periodically with PBS during the trough of every triangular wave to prevent dehydration. Native and de-epithelialized sample rings were taken from the mid-third of the trachea. The energy loss in joules (J) was calculated as the area of the fifth triangular wave of the load versus displacement curve and was performed in a mathematical programming environment (Matlab, The MathWorks, USA) using the trapezoidal rule.

### Live/dead assay

The area of live cartilage after the de-epithelialization protocol was evaluated using a staining kit for mammalian cells (Live/Dead® Viability/Cytotoxicity, Molecular Probes Inc., Canada) and the tissue samples were visualized on a laser scanning confocal microscope (10 × air magnification) (A1R, Nikon, Japan). Cartilage rings, native and de-epithelialized samples, were kept in DMEM supplemented with 10% (v/v) FBS and 1% (v/v) penicillin-streptomycin until the assay. For consistency purposes, 1–2 rings from fresh tracheae were kept in culture media for the same duration as the de-epithelialization protocol. Cartilage slices with a thickness of approximately 500 µm were incubated in 1.5 mL Eppendorfs tubes containing a dye solution of 1 mL PBS, 13 µL ethidium homodimer-1 red fluorescent, and 0.6 µL calcein-AM green fluorescent for 10–15 min. ImageJ was used to measure the percentage area of live chondrocytes compared to the total area of cartilage.

### *In vitro* cell seeding on de-epithelialized graft submucosa

BEAS-2B cells were seeded onto porcine tracheal submucosa in order to evaluate cell viability on the de-epithelialized tracheal lumen. De-epithelialized samples were harvested and stored in DMEM supplemented with 10% (v/v) FBS and 1% (v/v) penicillin-streptomycin at 4 °C until experimentation. The tracheal submucosa was shaved off the cartilage using a scalpel, cut into squares (8 × 8 mm); and placed individually onto 35 mm dishes. BEAS-2Bs, suspended in DMEM supplemented with 10% (v/v) FBS and 1% (v/v) penicillin-streptomycin, were seeded onto the samples at a cell density of 2 × 10^5^ cells/cm^2^ and incubated at 37 °C and 5% CO_2_. To ensure cell coverage of the submucosa, a 50 µL droplet of media containing the cells was first placed on top of submucosa squares. This droplet size was optimized to prevent dispersion of cells onto the rest of the dish during the initial 60 min of attachment. Once cells had formed the initial attachment, samples were then gently submerged and fully covered in media. Cell viability was assessed at 6 and 24 h after seeding. The Live/Dead® Viability/Cytotoxicity staining kit for mammalian cells (Molecular Probes, USA) was used with 4 µL ethidium homodimer-1 and 0.2 µL calcein-AM per 1 mL of PBS and placed onto the well for incubation at room temperature for 15 min. Cells were visualized using a two-photon/confocal microscope (LSM 710 NLO, Zeiss, Germany) and the acquired images were used for quantification and qualitative analysis. Cell viability was calculated as the percentage of live cells (stained with calcein-AM) per total cells (live and dead).

### Statistical analysis

Commercial computer statistical software (GraphPad Software Inc., USA) was used for statistical analysis and *p* values < 0.05 were considered significant. Descriptive statistics are presented as column bars with mean and standard deviation. Groups of two were analyzed with the two-tailed *t*-test and groups of three were evaluated for equal variance using Bartlett’s test for equal variance and subsequent one-way ANOVA with Tukey’s multiple comparison test.

## Supplementary information

Supplementary Information

## Data Availability

Data pertaining to PV curve and tensile test MATLAB codes are available upon request.
