# Supplementary material for: De-epithelialization of porcine tracheal allografts as an approach for tracheal tissue engineering
Source: Sci Rep. 2019 Aug 19;9:12034. doi: 10.1038/s41598-019-48450-4 (PMC6700109; doi:10.1038/s41598-019-48450-4)
Supplement: Supplementary file 1 — Supplementary Information [file 41598_2019_48450_MOESM1_ESM.docx]

**De-Epithelialization of porcine Tracheal allografts AS AN APPROACH FOR TRACHEAL TISSUE ENGINEERING**

Fabio G. Aoki, Ratna Varma, Alba E. Marin-Araujo, Hankyu Lee, John P. Soleas, Alexander H. Li, Kayla Soon, David Romero, Henrique T. Moriya, Siba Haykal, Cristina Amon, Thomas K. Waddell, and Golnaz Karoubi

**SUPPLEMENTARY RESULTS**

Histological analyses were performed to verify the efficiency of 1 % sodium dodecyl sulfate (SDS) in the removal of cells from the mucosa/submucosa observed with Haematoxylin and Eosin (H&E) staining and the effect on glycosaminoglycans (GAGs) with Alcian blue staining at different timepoints (Native, 1, 3, 5, 12, 24 and 48 h; Supplementary Fig. S1a). The 1 % SDS solution was able to remove cells from the mucosa/submucosa as early as 3 h after treatment. No evident histological changes were observed in cartilage up to the time of 48 h treatment with 1% SDS (Supplementary Fig. S1b).

Supplementary Fig. S2a shows a representative partially decellularized trachea before (Native) and after 3 and 24 h in 1 % SDS. There is a significant statistical difference for Native vs. 24 h of treatment in 1 % SDS in the mass and length (p < 0.05; Supplementary Fig. S2b, c) of tracheal graft. Based on these results, we chose to use the 3 h de-epithelialization timepoint in the bioreactor.

Cell debris from some deeper glands in samples obtained from the bioreactor de-epithelialization were not always completely removed (Supplementary Fig. S3).

Supplementary Fig. S4 presents the standard curves (performed in 3 experimental batches) used in quantification of sulfated glycosaminoglycans (sGAGs).

**SUPPLEMENTARY FIGURES**

**
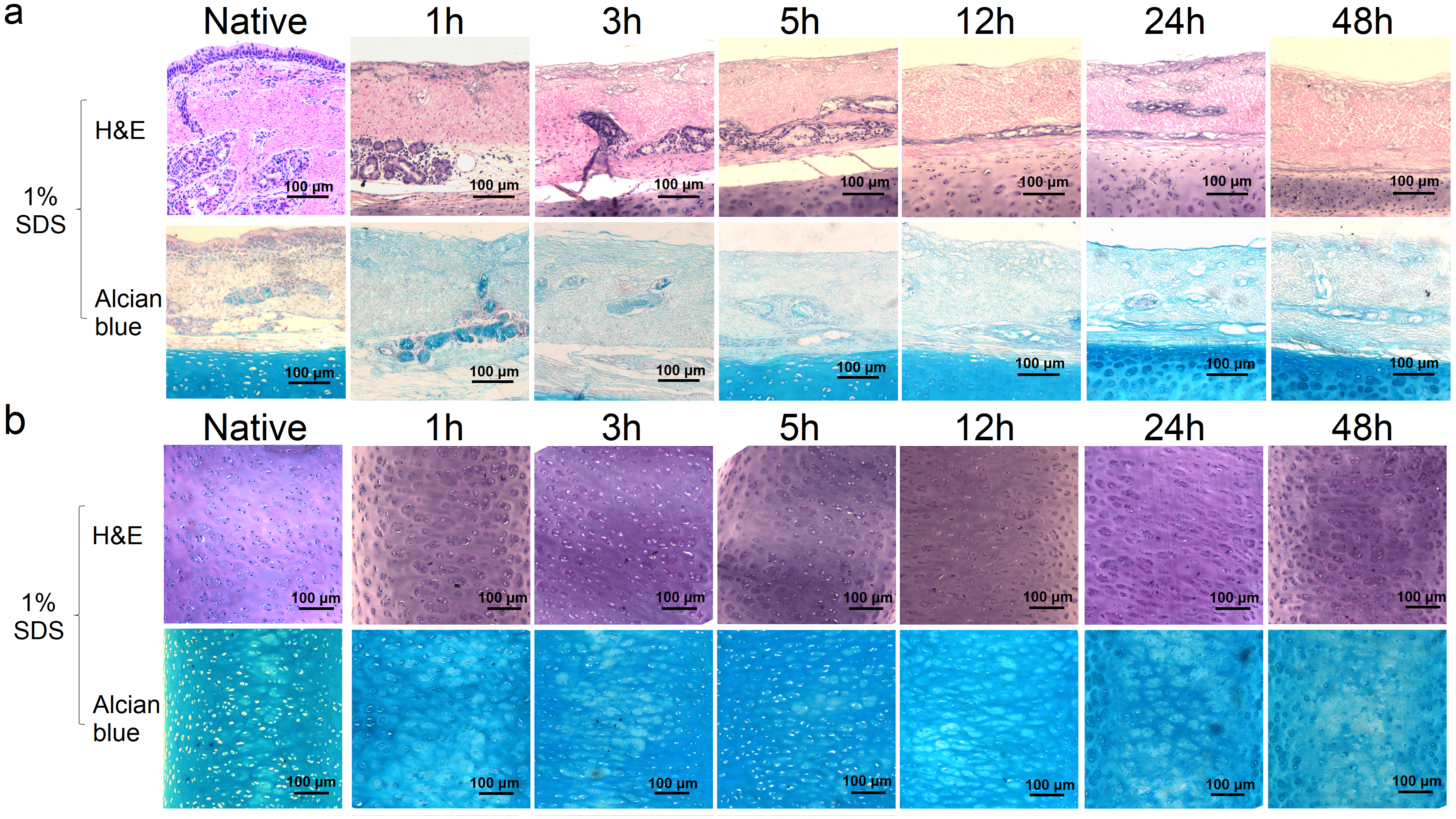
**

**Supplementary Figure S1. Haematoxylin and Eosin (H&E) and Alcian blue staining of partially-decellularized porcine tracheal grafts at different time points.**

Partial decellularization of tracheal samples via treatment with 1 % SDS solution (n = 3) at different time points (Native, 1, 3, 5, 12, 24 and 48 h). (a) Mucosa/Submucosa stained with H&E (top row) and Alcian blue staining for glycosaminoglycans (GAGs) (bottom row). (b) Cartilage rings stained with H&E (top row) and Alcian blue (bottom row).

**
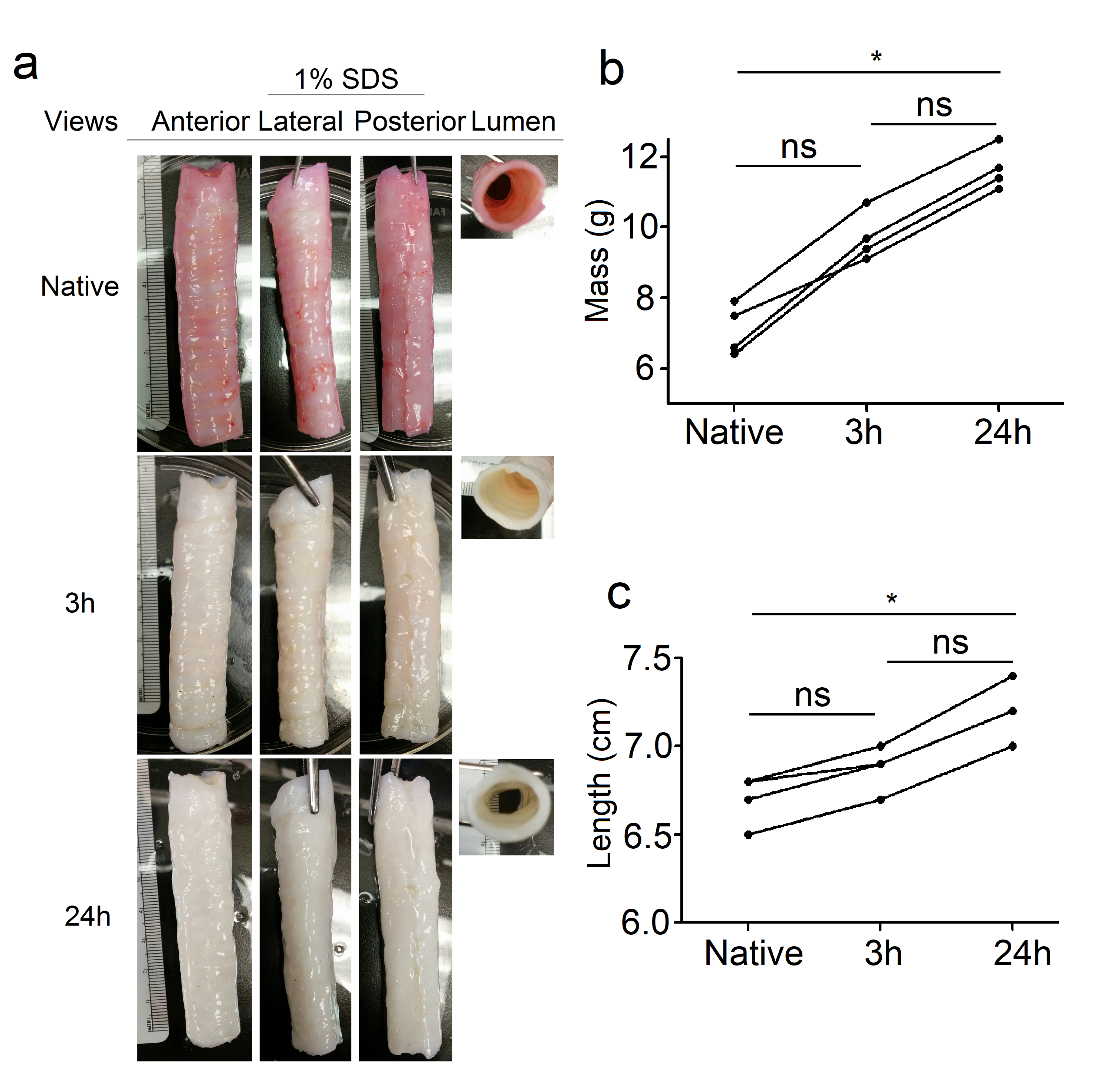
**

**Supplementary Figure S2. Partial decellularization of a trachea in 1 % SDS solution at different timepoints and measurements of the tracheal grafts.**

(a) Representative images of a tracheal sample before (Native) and after 3 and 24 h in 1 % SDS solution. Columns: different views of the same tracheal sample: anterior, lateral, posterior, and lumen, respectively. (b) Mass (g) and (c) length (cm) change before (Native) and after 3 and 24 h (n = 4) of decellularization protocol (Friedman's test followed by Dunn's *post hoc* comparison; ns, p > 0.05; *p < 0.05).


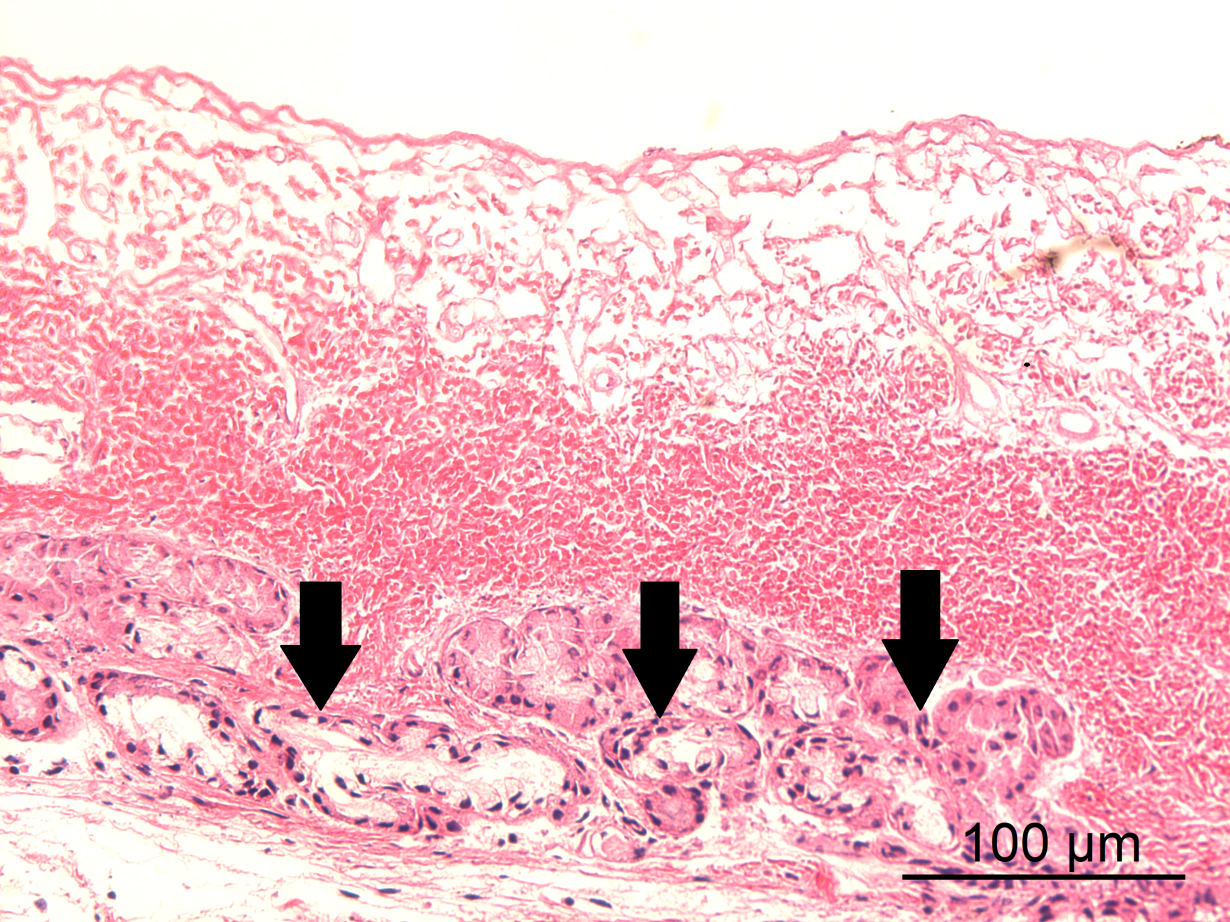


**Supplementary Figure S3. Haematoxylin and Eosin (H&E) staining showing incomplete gland decellularization.**

Representative brightfield microscopy image of de-epithelialized tracheal graft showing incomplete decellularization of submucosal gland (black arrows).

**Supplementary Figure S4. Standard curves used in quantification of sulfated glycosaminoglycans (sGAGs).**

**SUPPLEMENTARY METHODS**

*Partial Decellularization Protocol*

In order to select the shortest time interval for de-epithelialization, fresh tracheal samples were harvested and subjected to 1, 3, 5, 12, 24 and 48 h in 1 % SDS solution at 4 ºC in 50 mL Falcon tubes on a rocking platform at 60 rpm and washed 3 x 15 min in phosphate buffered saline (PBS).

*Trachea Morphometry*

Tracheal samples were photographed, weighed and the length was measured. Samples were obtained for histology. Histological samples were placed in tissue cassettes, fixed in 10 % formalin neutral buffered solution overnight and transferred to 70% ethanol until paraffin embedding. The paraffin blocks were sectioned (5 μm thickness) and slices placed onto positively charged slides, followed by deparaffinization in xylene. Samples obtained from partial decellularization at different time points were stained for H&E and Alcian blue.

**Supplementary Table S1. De-epithelialization protocol in the double-chamber bioreactor.**

| Step | Reagents | Time | | | Vol. (mL) | pH | | Temp. (°C) | |
| --- | --- | --- | --- | --- | --- | --- | --- | --- | --- |
| 1a | 1 % SDS | 3 h | | | 75 | 7.4 | | 37 | |
| 2b | diH2O | 40 min | | | 240 | - | | 37 | |
| 3b | 1 % Triton X-100 | 30 min | | | 140 | 7.4 | | 37 | |
| 4b | PBS | 30 min | | | 140 | 7.4 | | 37 | |
| The luminal side of the trachea (inner chamber) is treated step-wise with the listed reagents. The outside of the trachea (outer chamber) is bathed in culture media (DMEM supplemented with 10% FBS, 1% Pen/Strep). | | | | | | | | | |
| a - De-epithelialization process - same solution recirculates in the luminal circuit. | | | | | | | | | |
| b - Washing steps - one-way flow. | | |  |  | | |  | |  |
